# Supplementary material for: Urothelial organoids originating from Cd49fhigh mouse stem cells display Notch-dependent differentiation capacity
Source: Nat Commun. 2019 Sep 27;10:4407. doi: 10.1038/s41467-019-12307-1 (PMC6764959; doi:10.1038/s41467-019-12307-1)
Supplement: Supplementary file 1 — Supplementary information_new [file 41467_2019_12307_MOESM1_ESM.pdf]

## Supplementary Information - Santos et al.

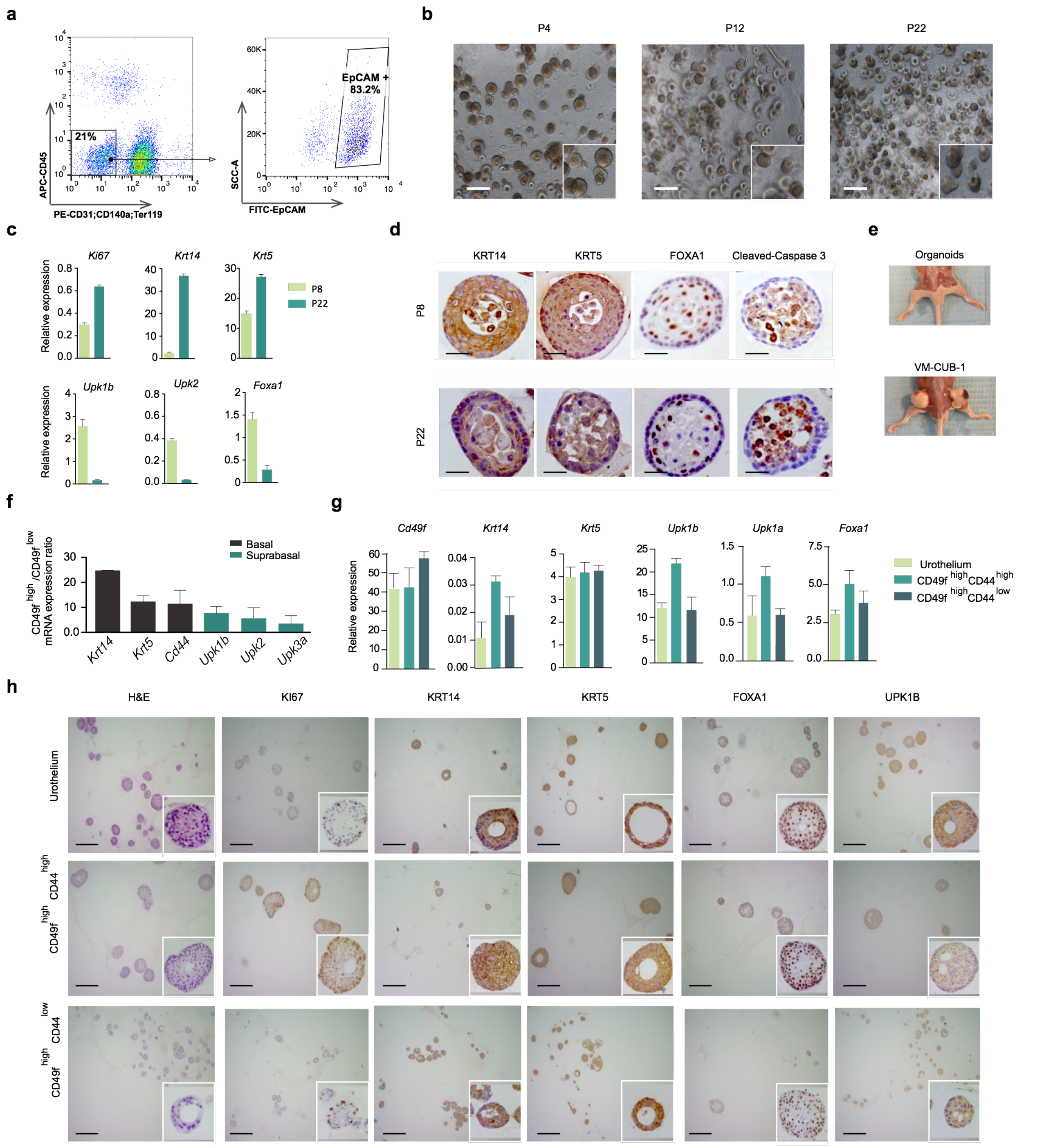


**Supplementary Figure 1. CD49f labels an organoid-forming urothelial cell population with stem cell features.** (**a**) Flow cytometry analysis reveals that urothelial digests contain ~20% EpCAM$^+$ epithelial cells with a major contribution of fibroblasts, leukocytes and endothelial cells. (**b**) Representative phase contrast images of organoids at different passages (P4, P12 and P22) (scale bar, 500μm). (**c**) RT-qPCR analysis of the expression of urothelial differentiation genes in proliferative (P) organoids at P8 and P22. Continued culture was associated with an enrichment of cells expressing basal markers. (**d**) Immunohistochemical analysis of urothelial differentiation marker expression in P organoids at P8 and P22 (scale bar, 100μm). (**e**) Subcutaneous xenotransplantation in the flank of nude mice; VM-CUB-1 urothelial bladder cancer cells were used as a positive control (*n*=6). (**f**) Relative enrichment of urothelial marker transcripts in CD49f$^{high}$ cells compared to CD49f$^{low}$ cells. Graph representative of one experiment out of 2; error bars indicate SD. (**g**) RT-qPCR analysis of the expression of urothelial differentiation markers in organoids derived from FACS-sorted CD49f$^{high}$ CD44$^{high}$ and CD49f$^{high}$ CD44$^{low}$ cells compared to those derived from unsorted cells; error bars indicate SEM. (**h**) H&E and immunohistochemical analysis of Ki67 and urothelial differentiation markers in organoids generated from the indicated FACS populations (scale bar, 750μm).

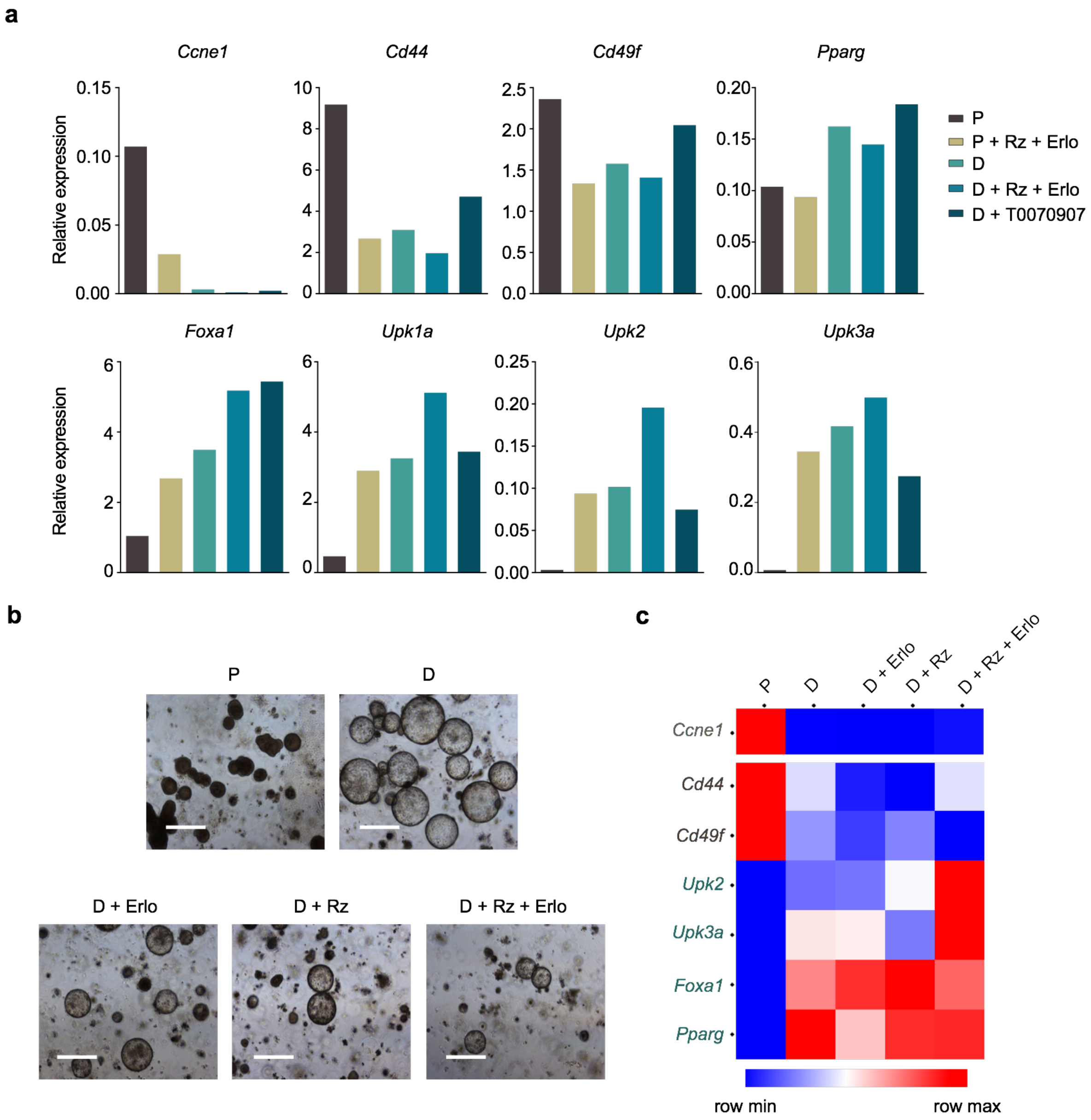


**Supplementary Figure 2. Pharmacological modulation of EGFR and PPARγ activity potentiates organoid differentiation.** (**a**) RT-qPCR expression analysis of cell cycle and canonical urothelial differentiation markers in proliferative (P) or differentiated (D) organoids treated with Rz+Erlotinib, and with the PPARγ inverse agonist T0070907 (n=2). Data were normalized to *Hprt* expression. (**b**) Phase contrast images of control (P, D) and drug-treated organoids (scale bar, 500 μm). (**c**) Heatmap representing the expression of proliferation and urothelial differentiation markers in control and drug-treated organoids. P, proliferation; D, differentiation. Er, erlotinib; Rz, rosiglitazone.

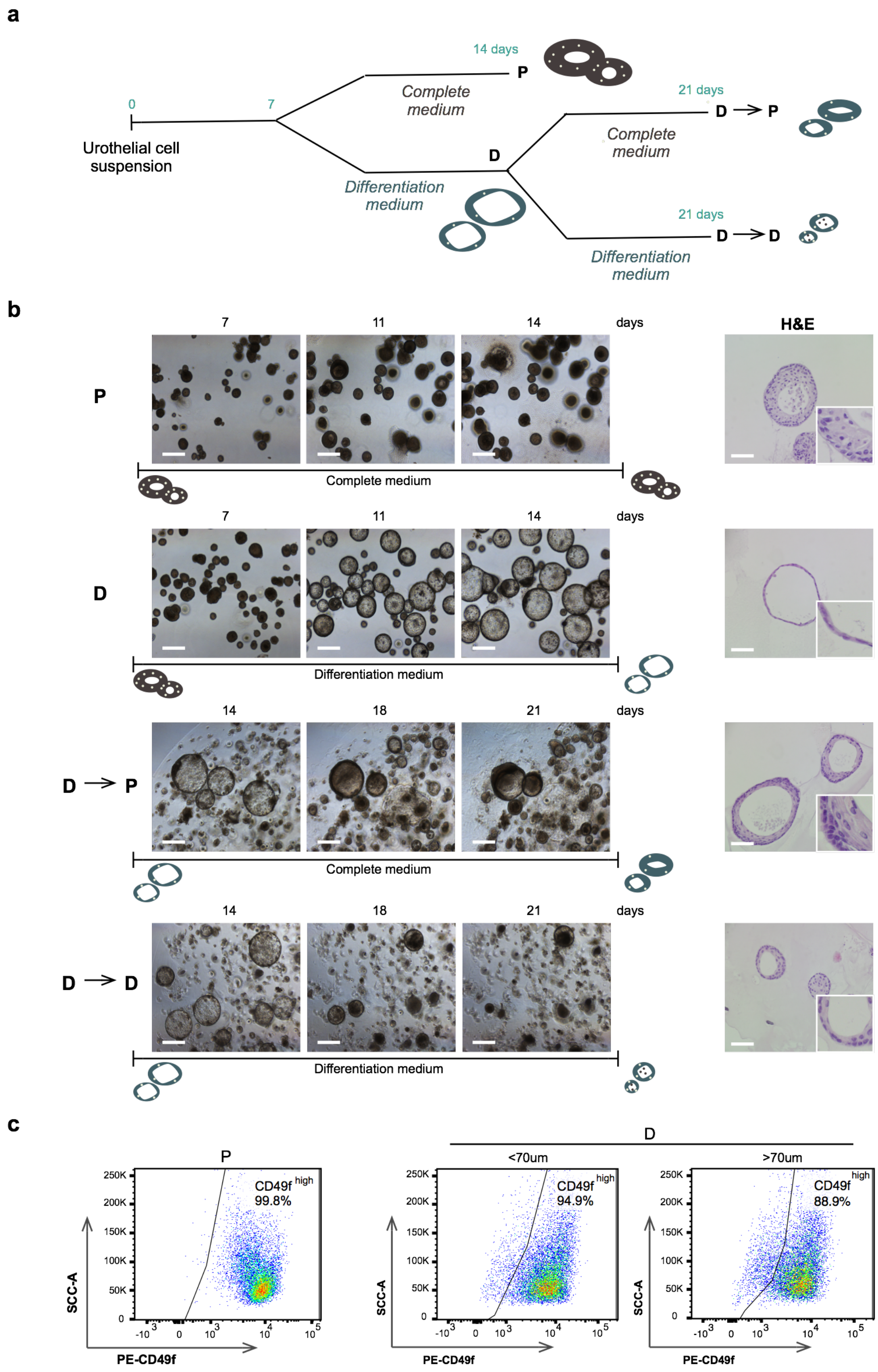


**Supplementary Figure 3. Differentiated (D) organoids are able to re-enter the cell cycle upon exposure to CM.** (**a**) Experimental design to assess the ability of D organoids to respond to growth factor stimulation. (**b**) Representative phase contrast images of organoids cultured as described in (a) and H&E staining of organoids exposed to complete medium or differentiation medium at day 7 and/or 21 (scale bars 500 µm and 250 µm, respectively). (**c**) After Matrigel removal, proliferative (P) organoids were fractionated according to size using a 70 µm filter, re-embedded in Matrigel, and cultured for an additional 7 days in differentiation medium. Representative flow cytometry plots showing CD49f expression in cells dissociated from P and D organoids (D<70 µm and D>70 µm) (*n*=2).

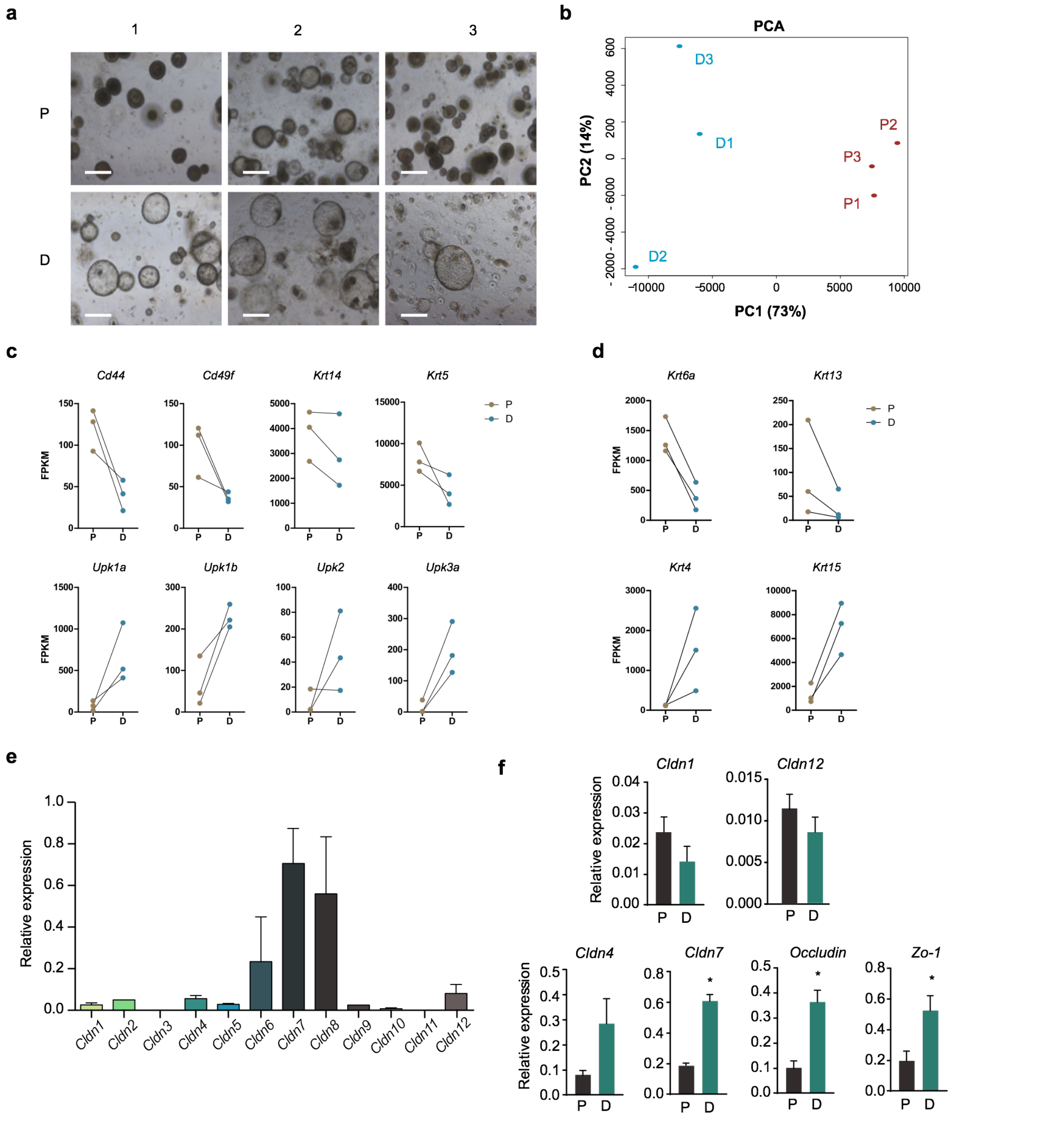


**Supplementary Figure 4. Transcriptome analysis reveals the differentiation capacity of organoids and identifies pathways activated during differentiation.** (**a**) Phase contrast images of proliferative (P) and differentiated (D) organoids used for RNA-seq (n=3) (scale bar, 500μm). (**b**) Principal component analysis of the transcriptomes of organoids used for RNA-seq showing greater divergence in differentiated conditions. (**c**) mRNA expression (FPKM values) of basal (top) and luminal/urothelial (bottom) markers in paired samples of P and D organoids showing evidence of urothelial differentiation. (**d**) mRNA expression (FPKM values) of novel keratin mRNA species with significant differential expression in P and D organoids, providing new markers for to assess the hierarchical urothelial differentiation program. (**e**) RT-qPCR analysis of claudin transcripts in normal mouse urothelium. Data are normalized to *Hprt* expression. (**f**) Validation of expression of transcripts coding for tight junction components in P and D organoids. Relative expression to *Hprt* (Mann-Whitney test, error bars SD).

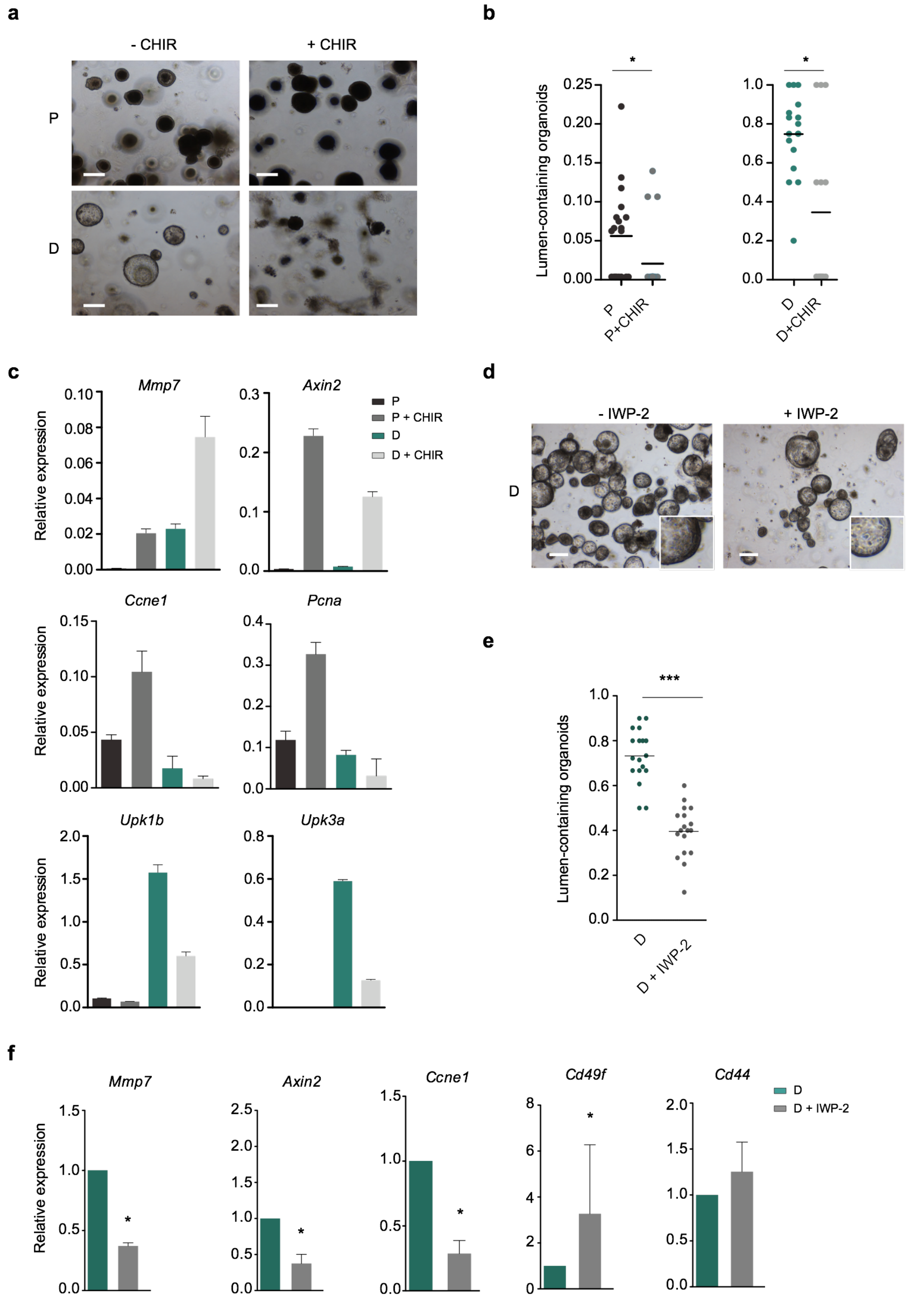


**Supplementary Figure 5. Effect of Wnt agonist CHIR99021 on proliferative (P) and differentiated (D) organoids** (n=1 experiment). **(a)** Phase contrast images (scale bar, 500 μm). **(b)** Quantification of lumen formation. **(c)** RT-qPCR analysis of the expression of Wnt target genes, proliferation and urothelial differentiation markers in P and D organoids treated with CHIR99021. Data are normalized to *Hprt* expression. (**d-f**) Effect of the Wnt processing inhibitor IWP-2 on differentiated organoids (n=3). (**d**) Phase contrast images (scale bar, 500μm). **(e)** Quantification of lumen formation. (**f**) Expression analysis of Wnt target genes and urothelial basal markers using RT-qPCR. Error bars indicate SEM; * p ≤ 0.05, ** p ≤ 0.01; *** p ≤ 0.001.

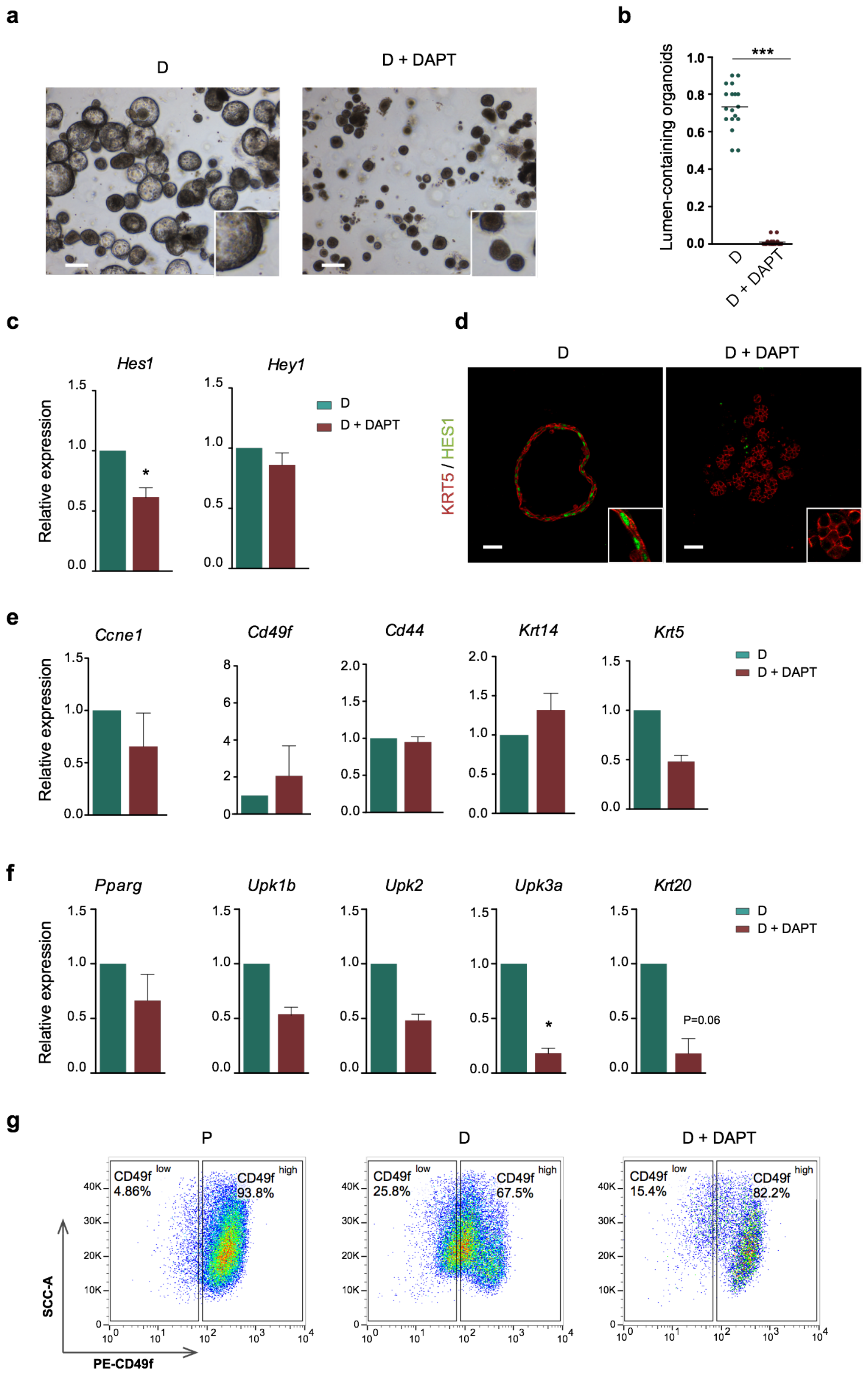


**Supplementary Figure 6. Effect of the pharmacological inhibition of Notch, using the gamma secretase inhibitor DAPT, on urothelial organoid renewal and differentiation** (n=5). (**a**) Phase contrast images (scale bar, 500µm). (**b**) Quantification of lumen formation. (**c**, **d**) Expression analysis of Notch target genes using (**c**) RT-qPCR and (**d**) immunofluorescence (scale bar, 250 µm). (**e**) RT-qPCR analysis of expression of proliferation and basal urothelial markers in differentiated organoids treated with DAPT. (**f**) RT-qPCR analysis of urothelial markers in differentiated organoids treated with DAPT. (**g**) FACS plots depicting the analysis of CD49f$^{low}$ and CD49f$^{high}$ cell populations in differentiated organoids treated with DAPT. Error bars indicate SEM. * p ≤ 0.05, ** p ≤ 0.01; *** p ≤ 0.001.

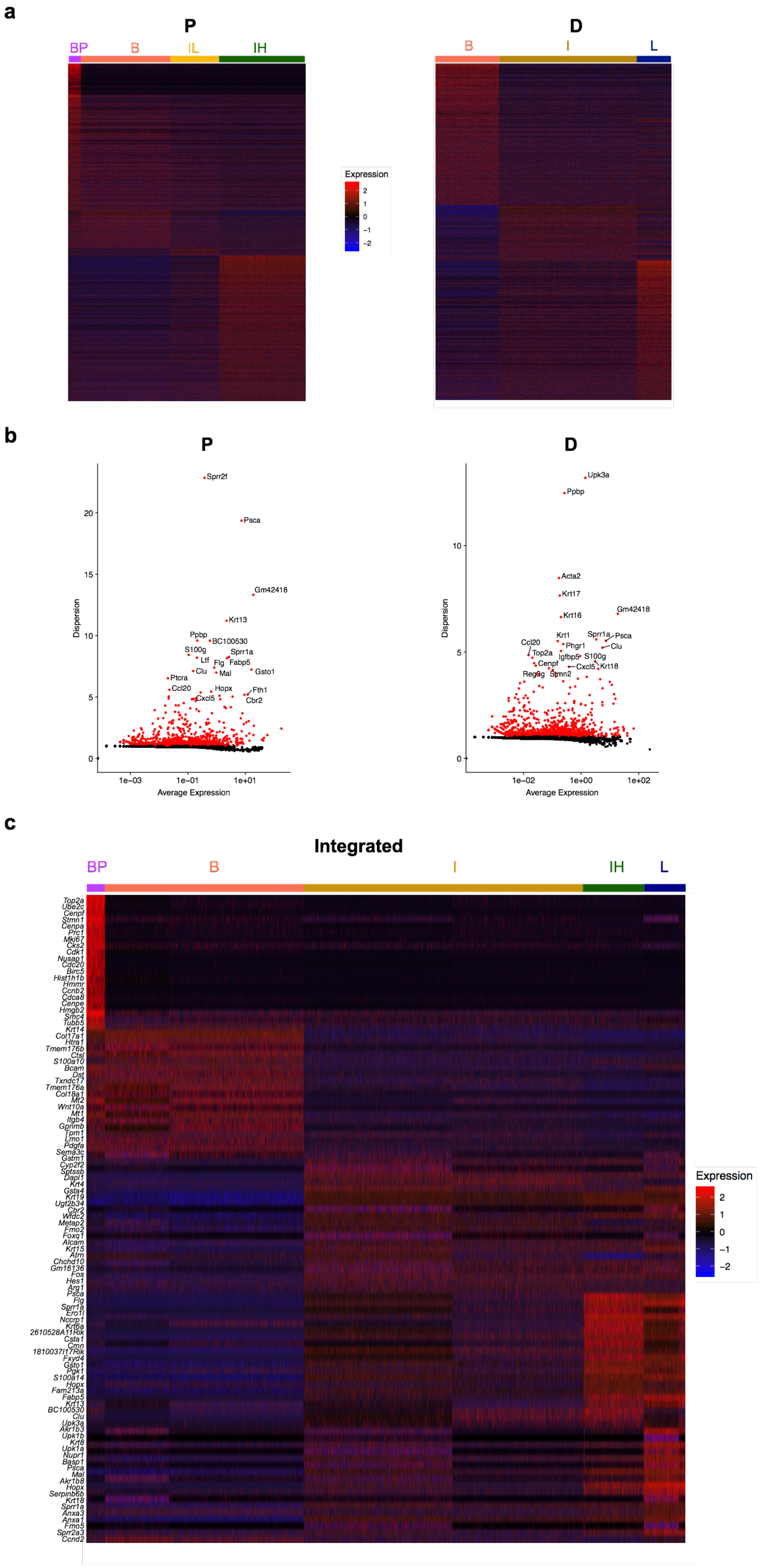


**Supplementary Figure 7. Single cell transcriptomic analysis of urothelial organoids.** (**a**) Heatmap displaying the expression of the cell cluster markers identified as DEGs in proliferative (P) and differentiated (D) organoids with a log-fold change >0.25 and <0.05 FDR. Markers were identified by differential expression analysis (Wilcoxon Rank Sum test). (**b**) Highly variable genes expressed in P vs. D organoids (2000 selected genes, in red) (Supplementary Data 2). **(c**) Heatmap displaying the expression of the top 20 genes differentially identified as significant cell cluster markers in the integrated analysis. For P organoids: BP (Basal-Proliferative), B (Basal), IL (Intermediate-Low) and IH (Intermediate-High); for D organoids: B (Basal), I (Intermediate) and L (Luminal); for Integrated: BP (Basal Proliferative), B (Basal), I (Intermediate), IH (Intermediate High) and L (Luminal).

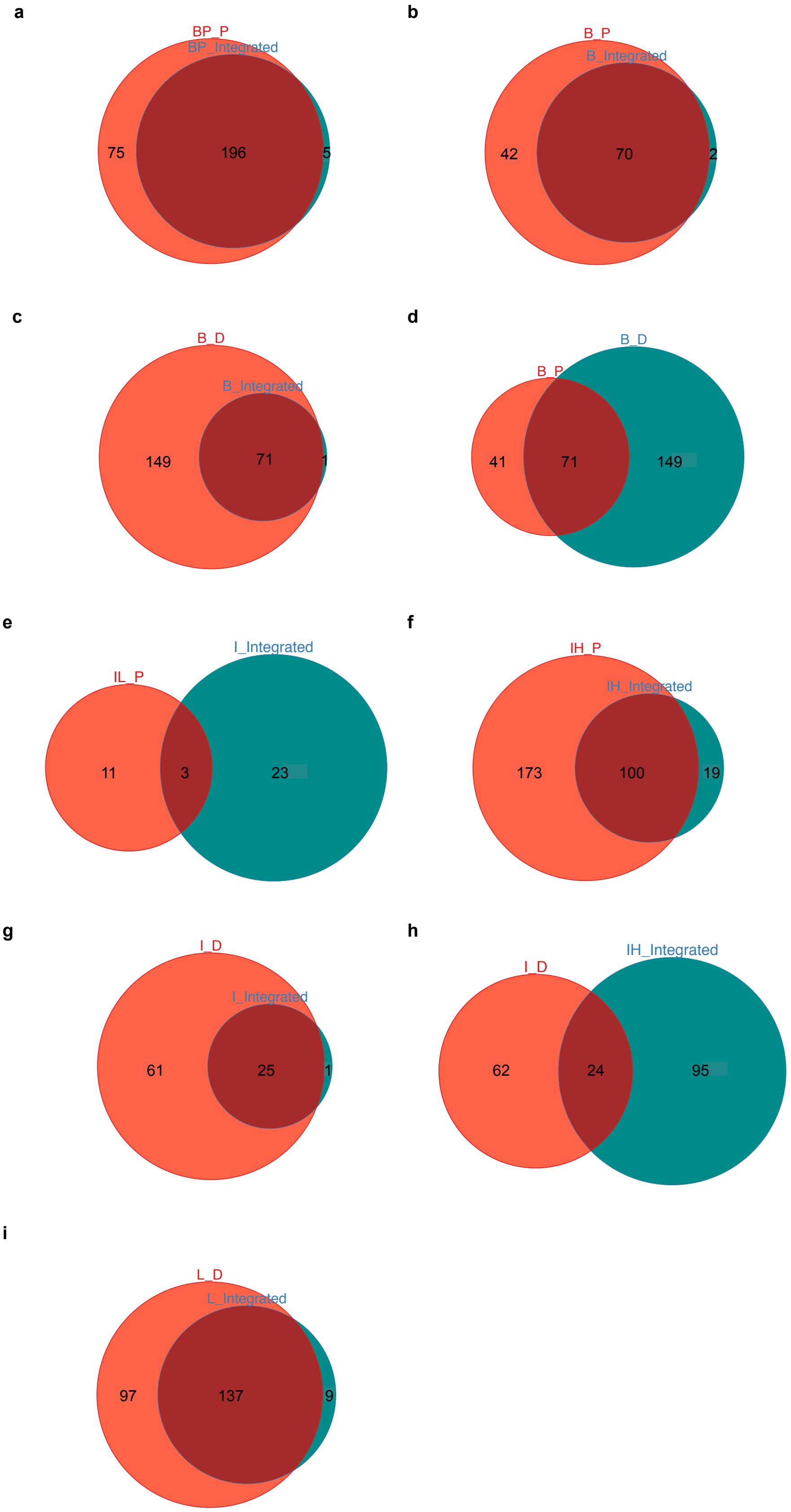


**Supplementary Figure 8. Venn diagrams showing the overlap of cluster markers identified through individual and integrated analyses.** (**a**) Basal-Proliferative P versus Basal-Proliferative Integrated. (**b**) Basal P versus Basal Integrated. (**c**) Basal D versus Basal Integrated. (**d**) Basal P versus Basal D. (**e**) Intermediate-Low P versus Intermediate Integrated. (**f**) Intermediate-High P versus Intermediate-High Integrated. (**g**) Intermediate D versus Intermediate Integrated. (**h**) Intermediate D versus Intermediate high Integrated. (**i**) Luminal D versus Luminal Integrated. P, proliferative organoids; D, differentiated organoids.

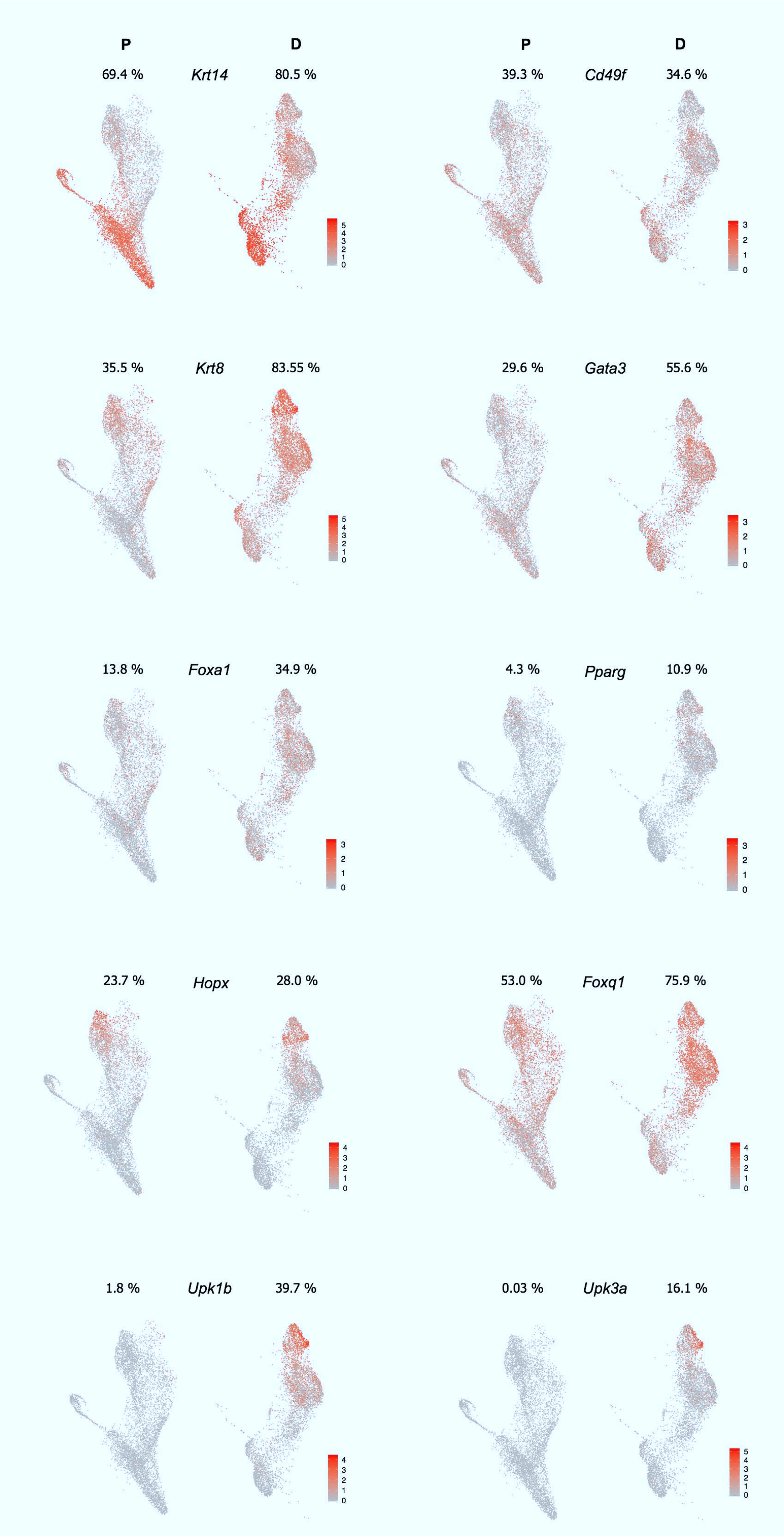


**Supplementary Figure 9.** UMAP plots derived from the split Integrated analysis showing the expression of selected basal (***Krt14*** and ***Cd49f***), intermediate (***Krt8***), and luminal (***Upk1b*** and ***Upk3a***) markers, three well-established transcription factors involved in urothelial differentiation (*Gata3*, *Foxa1*, *Pparg*), and newly identified candidate transcription factors (*Hopx, Foxq1*). The percentage of cells in which each of the markers was detected is shown.

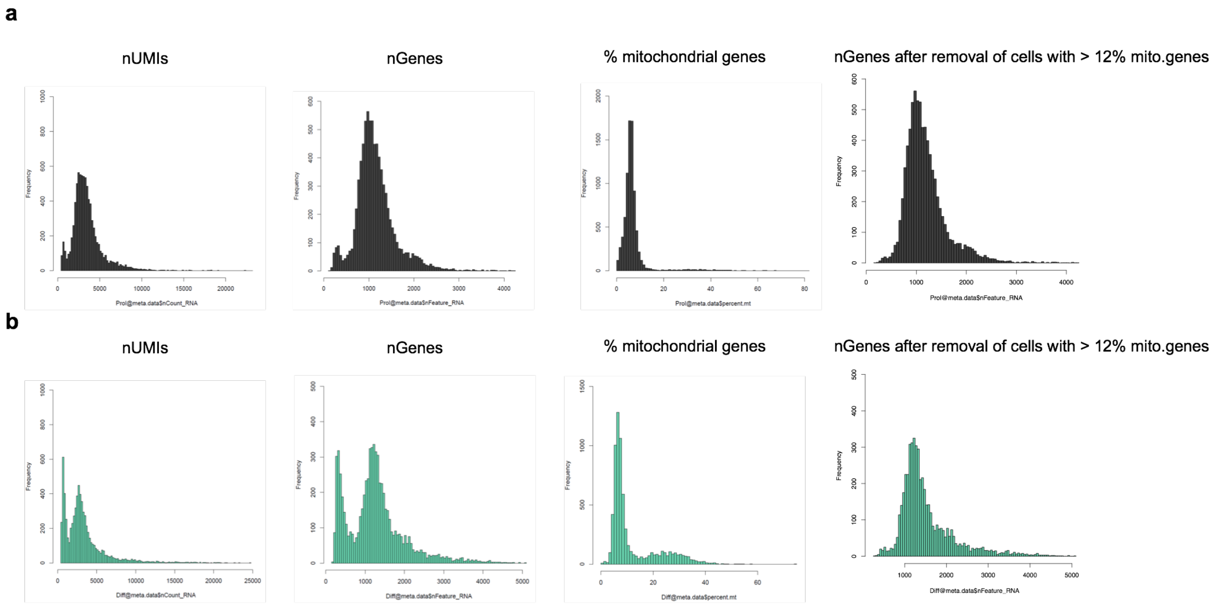


**Supplementary Figure 10. Single-cell RNA-Seq data pre-processing.** Histograms showing the distribution of the number of unique molecular identifiers (nUMIs), genes (nGenes), percentage of mitochondria-encoded genes (% mitochondrial genes), and genes remaining after removal of cells expressing >12% of mitochondria-encoded genes for proliferative (**a**) and differentiated (**b**) datasets.

**Supplementary Table 1. Sequences of the primers used for qRT-PCR.**

| Gene name | Forward primer (5'-3') | Reverse primer (5'-3') |
|---|---|---|
| *Axin2* | GAGAGTGAGCGGCAG AGC | CGGCTGACTCGTTCTCCT |
| *Cd44* | TGCATTTGGTGAACAAGGAA | GGAATGACGTCTCCAATCGT |
| *Cldn1* | AGGTCTGGCGACATTAGTGG | CGTGGTGTTGGGTAAGAGGT |
| *Cldn2* | GTGGCTGTAGTGGGTGGAGT | CCAAAGAAAACAGGGCTGAG |
| *Cldn3* | GCACCCACCAAGATCCTCTA | AGCCTGTCTGTCCTCTTCCA |
| *Cldn4* | ATGGCGTCTATGGGACTACA | TTACACATAGTTGCTGGCGG |
| *Cldn5* | CCTTCCTGGACCACAACATC | GCCGGTCAAGGTAACAAAGA |
| *Cldn6* | GTCCTGTGTGGTTCAGAGCA | GATGATGCCAGAGATGAGCA |
| *Cldn7* | TTTCATTGTGGCAGGTCTTG | CCAGAAGGACCAGAGCAGAC |
| *Cldn8* | TCCTCGGAATGAAGTGCACC | GCTGACAGGGATGAGCACAA |
| *Cldn9* | AACTCCAACACAGTGCTCCC | GGTCATGCCGAGGAGTTCAA |
| *Cldn10* | GGCTGTTCCCTGTATGCAAAC | TCTCCGCCTTGATACTTGGTC |
| *Cldn11* | CTGGTGGACATCCTCATCCT | ACCCAAGTCAGCAATGTTCC |
| *Cldn12* | GCTCCTGTTGCATCTGAGTTC | GGTAATCAGCGTTTCTCCAAGC |
| *Ccne1* | TGTTTTTGCAAGACCCAGATGA | GGCTGACTGCTATCCTCGCT |
| *Foxa1* | TGGAGTTCATAGAGCCCAGG | CATGAGAGCAACGACTGGAA |
| *Hes1* | TCCAAGCTAGAGAAGGCAGACA | CGTTCATGAACTCGCTGAA |
| *Hey1* | AAAGACGGAGAGGCATCATCG | TCCCTGCTTCTCAAAGGCACT |
| *Itga6/Cd49f* | AGTGCTTCTGCCCGAGGT | GGAGCCTCTTCGGCTTCTC |
| *Ki67* | ATCATTGACCGCTCCTTTAGGT | GCTCGCCTTGATGGTTCCT |
| *Krt5* | ACATTAACAACCTCCGTAGACAG | CGCTTGTTGATCTCATCCTC |
| *Krt14* | AGGTGAAGATTCGGGACTGG | GCTCCGTCTCAAACTTGGTC |
| *Krt20* | CCTTGGAGATCAGCTTCCAC | CCTGCGAATTGACAATGCTA |
| *Mmp7* | CGGAGATGCTCACTTTGACA | CCAGAGAGTGGCCAAATTCA |
| *Ocldn* | CCTCCAATGGCAAAGTGAAT | CTCCCCACCTGTCGTGTAGT |
| *Pcna* | CGCAGAGGGTTGGTAGTTGT | TGGACATGCTGGTGAGGTTC |
| *Pparg* | CGCTGGGGTATTGGGTCG | TTCAAATCTTGTCTGTCACACAGT |
| *Upk1a* | GGCCTGACAGCAAAATAATGA | GAGAAGCAGGAAGATGGCTT |
| *Upk1b* | AATCAACAGGCCCTGGAAG | GAAGAAGGCAGAGGAGACCA |
| *Upk2* | GACAGCAGACCAGAGAGGCT | ACACTGCCTGTCCAGACCTT |
| *Upk3a* | AGTAGTGCTCAGTGGGACGC | AGCGGCTCTTACGAGGTTTA |
| *Zo1* | CCACCTCTGTCCAGCTCTTC | CACCGGAGTGATGGTTTTCT |
