# Supplementary material for: Urothelial organoids originating from Cd49fhigh mouse stem cells display Notch-dependent differentiation capacity
Source: Nat Commun. 2019 Sep 27;10:4407. doi: 10.1038/s41467-019-12307-1 (PMC6764959; doi:10.1038/s41467-019-12307-1)
Supplement: Supplementary file 5 — Reporting Summary [file 41467_2019_12307_MOESM5_ESM.pdf]

nature research

Corresponding author(s): Francisco X Real

Last updated by author(s): 2019/08/07

Reporting Summary

Nature Research wishes to improve the reproducibility of the work that we publish. This form provides structure for consistency and transparency in reporting. For further information on Nature Research policies, see Authors & Referees and the Editorial Policy Checklist.

Statistics

For all statistical analyses, confirm that the following items are present in the figure legend, table legend, main text, or Methods section.

n/a	Confirmed	
☐	☒	The exact sample size (*n*) for each experimental group/condition, given as a discrete number and unit of measurement
☐	☒	A statement on whether measurements were taken from distinct samples or whether the same sample was measured repeatedly
☐	☒	The statistical test(s) used AND whether they are one- or two-sided<br>*Only common tests should be described solely by name; describe more complex techniques in the Methods section.*
☒	☐	A description of all covariates tested
☐	☒	A description of any assumptions or corrections, such as tests of normality and adjustment for multiple comparisons
☐	☒	A full description of the statistical parameters including central tendency (e.g. means) or other basic estimates (e.g. regression coefficient) AND variation (e.g. standard deviation) or associated estimates of uncertainty (e.g. confidence intervals)
☒	☐	For null hypothesis testing, the test statistic (e.g. *F*, *t*, *r*) with confidence intervals, effect sizes, degrees of freedom and *P* value noted<br>*Give P values as exact values whenever suitable.*
☒	☐	For Bayesian analysis, information on the choice of priors and Markov chain Monte Carlo settings
☒	☐	For hierarchical and complex designs, identification of the appropriate level for tests and full reporting of outcomes
☒	☐	Estimates of effect sizes (e.g. Cohen's *d*, Pearson's *r*), indicating how they were calculated

Our web collection on statistics for biologists contains articles on many of the points above.

Software and code

Policy information about availability of computer code

Data collection

- Flow cytometry- FACS Influx or Ariall (BD Biosciences) flow cytometer
- Barrier function assays- 40x immersion oil objective (HCX PLAPO 1.2 NA)- TCS-SP5 (AOBS) Leica microsystem laser scanning confocal microscope
- Organoid quantification- CCD-microscope using a bright-field filter
- Organoid video- HC PL APO 10x 0.4N.A dry objective- DMI6000B bright field microscope, Leica Microsystems
- scRNA-seq- Chromium Single Cell 3′ GEM, Library & Gel Bead Kit v3, PN-1000075; NextSeq500 instrument (Illumina)

Data analysis

- Immmunofluorescence quantification - Definiens XD v2.5 software
- Flow cytometry analysis - FlowJo version 10.2, LLC.
- RNA-Seq - Illumina Real Time Analysis software (RTA1.13); Nextpresso version 1.9.1
- scRNA-seq - bcl2fastq 2.19.0, Illumina; Cell Ranger version 3.0.2 software; Seurat R package (version 3.0.0.9000); SC3 R package

For manuscripts utilizing custom algorithms or software that are central to the research but not yet described in published literature, software must be made available to editors/reviewers. We strongly encourage code deposition in a community repository (e.g. GitHub). See the Nature Research guidelines for submitting code & software for further information.

Data

Policy information about availability of data

All manuscripts must include a data availability statement. This statement should provide the following information, where applicable:

- Accession codes, unique identifiers, or web links for publicly available datasets
- A list of figures that have associated raw data
- A description of any restrictions on data availability

There are no restrictions on the availability of our materials or data.

The bulk RNA-seq data generated in this study have been deposited in GEO with accession number GSE109566.
The single-cell RNA-Seq data have been deposited in GEO with accession number GSE131909.

Field-specific reporting

Please select the one below that is the best fit for your research. If you are not sure, read the appropriate sections before making your selection.

☒ Life sciences ☐ Behavioural & social sciences ☐ Ecological, evolutionary & environmental sciences

For a reference copy of the document with all sections, see nature.com/documents/nr-reporting-summary-flat.pdf

Life sciences study design

All studies must disclose on these points even when the disclosure is negative.

Sample size	No previous sample size calculation was used. The number of mice used was based on the previous experience of the laboratory in using these assays and mouse strains.<br>In selected cases, a pilot experiment was performed to decide the final number of mice to be used.<br>For selected experiments (i.e. RNA-Seq) a "feasible" number of mice was used that was estimated to provide sufficient statistical power.
Data exclusions	No data were excluded from the analysis.
Replication	For all major experiments, at least two - and generally 3 - experiments were performed.
Randomization	Samples were allocated to experimental group without any previous selection.
Blinding	Samples were coded. Experiments were performed and then data were decoded. The code was unknown to the investigator at the time of analysis.

Reporting for specific materials, systems and methods

We require information from authors about some types of materials, experimental systems and methods used in many studies. Here, indicate whether each material, system or method listed is relevant to your study. If you are not sure if a list item applies to your research, read the appropriate section before selecting a response.

Materials & experimental systems

n/a	Involved in the study	
☐	☒	Antibodies
☐	☒	Eukaryotic cell lines
☒	☐	Palaeontology
☐	☒	Animals and other organisms
☒	☐	Human research participants
☒	☐	Clinical data

Methods

n/a	Involved in the study	
☒	☐	ChIP-seq
☐	☒	Flow cytometry
☒	☐	MRI-based neuroimaging

Antibodies

Antibodies used

For FACS: FITC-labeled anti-mouse EpCAM 1:100 Bio Legend; APC-labeled anti-mouse EpCAM 1:100 Bio Legend; FITC-labeled WGA 1: 1250Vector Laboratories; PE-labeled anti-mouse/human CD49f 1:500 BD Biosciences; APC-labeled anti-mouse CD45 1:100 BD biosciences; PE-labeled anti-mouse CD31 1:100 BD biosciences; PE-labeled anti-mouse CD140a 1:100 Labclinics; PE-labeled anti-mouse Ter119 1:100 BioLegend; APC-labeled anti-mouse/human CD44 1:300 BioLegend.

For WB: mouse monoclonal anti-UPK3a 1:5000 Santa Cruz: mouse monoclonal anti-UPK1b 1:5000 Dr. A. García-España; goat polyclonal anti-Lamin 1:5000 Santa Cruz; rabbit monoclonal anti-HES1 1:1000 Dr T. Sudo; mouse monoclonal anti-TP63 1:5000 Abcam.

For IF: Alexa Fluor 488-labeled goat anti-mouse Ig 1:200 Life Technologies; Alexa Fluor 555-labeled goat anti-rabbit Ig 1:200 Life Technologies; Alexa Fluor 488-labeled goat anti-chicken Ig 1:200 Life Technologies; Alexa Fluor 680-labeled goat anti-rabbit Ig 1:200 Life Technologies; FITC-labeled WGA 1:100 Vectorlabs; PE-labeled anti-mouse/human CD44 1:50 BioLegend; PE-labeled anti-mouse/human CD49f 1:100 BD Biosciences; Mouse monoclonal anti-Claudin4 1:100 Santa Cruz; Rat monoclonal; Rat monoclonal anti-Zo-1 1:100 Santa Cruz; Rabbit monoclonal anti-PPARg; 1:100 Cell Signalling; Rabbit polyclonal anti-Ki67 1:400 Novocastra; Rabbit monoclonal anti-FOXA1 1:100 Abcam; Mouse monoclonal anti-E-Cadherin 1:700 BD Biosciences; Rabbit monoclonal anti-Cleaved-caspase3 1:100 Cell Signalling; Rabbit polyclonal anti-KRT5 1:3000 BioLegend; Rabbit polyclonal anti-KRT14 1:3000 BioLegend; Rabbit polyclonal anti-TP53 1:100 Abcam; mouse monoclonal anti-UPK3a 1:100 Santa Cruz; mouse monoclonal anti-UPK1b 1:200 Dr. A. García-España; rabbit monoclonal anti-ITGA6/CD49f 1:100 Abcam; rat monoclonal anti-HES1 1:100 CNIO Monoclonal Antibody Core Unit.

Validation

A list with all antibodies used and antibody source and dilution is provided.
Validation was performed following variable strategies: cellular distribution, size of bands in western blotting experiments; for critical antibodies, control tissues were used for validation.

Eukaryotic cell lines

Policy information about cell lines

Cell line source(s)

ScaBER (as specified in Earl et al. PMID 25997541)
RT112 (as specified in Earl et al. PMID 25997541)
VM-CUB-1 (as specified in Earl et al. PMID 25997541)
RT4 (as specified in Earl et al. PMID 25997541)
LWnt3a, from Dr. E. Batlle (IRB Barcelona, Spain)
Expi293 RSPO-1 Life Technologies
RAW 264.7 Dr. M. Soengas (CNIO, Madrid, Spain)

Authentication

As specified in Earl et al. PMID 25997541

Mycoplasma contamination

Yes, tested and only Mycoplasma-negative cultures were used.

Commonly misidentified lines
(See ICLAC register)

No commonly misidentified cell lines were used.

Animals and other organisms

Policy information about studies involving animals; ARRIVE guidelines recommended for reporting animal research

Laboratory animals

Information provided in detail in the Methods section.
C57BL/6 000664 Jackson Laboratories.
129-Gt.ROSA26 Sor-CAG-EGFPLuo 006053 Jackson Laboratories.
ROSA26 mT/mG 007576 Jackson Laboratories.
Hsd:Athymic Nude-Foxn1nu 002019 Jackson Laboratories.

Wild animals

Provide details on animals observed in or captured in the field; report species, sex and age where possible. Describe how animals were caught and transported and what happened to captive animals after the study (if killed, explain why and describe method; if released, say where and when) OR state that the study did not involve wild animals.

Field-collected samples

For laboratory work with field-collected samples, describe all relevant parameters such as housing, maintenance, temperature, photoperiod and end-of-experiment protocol OR state that the study did not involve samples collected from the field.

Ethics oversight

Identify the organization(s) that approved or provided guidance on the study protocol, OR state that no ethical approval or guidance was required and explain why not.

Note that full information on the approval of the study protocol must also be provided in the manuscript.

Flow Cytometry

Plots

Confirm that:

☒ The axis labels state the marker and fluorochrome used (e.g. CD4-FITC).

☒ The axis scales are clearly visible. Include numbers along axes only for bottom left plot of group (a 'group' is an analysis of identical markers).

☒ All plots are contour plots with outliers or pseudocolor plots.

☒ A numerical value for number of cells or percentage (with statistics) is provided.

Methodology

Sample preparation

Urothelial cell suspensions obtained as previously described were incubated with blocking buffer (1% BSA/3mM EDTA in PBS) for 15 min at room temperature. After washing twice with PBS, cells were incubated with the primary antibodies in FACS buffer (0.1%BSA/3mM EDTA in PBS) for 30 min at 4 ºC. After washing twice with PBS, cells were resuspended in FACS buffer and stained with DAPI. In the experiments using isolated urothelial cells, EpCAM+ single cells were sorted by FACS and dead cells were excluded from subsequent analyses. In the experiments with NMU-o, samples were disaggregated as described in the methods section.

Instrument	FACS Influx or AriaII (BD Biosciences) flow cytometer.
Software	FlowJo version 10.2, LLC.
Cell population abundance	In all the experiments, a control sample lacking primary antibody and a Fluorescence Minus One (FMO) control were used and at least 10,000 events were acquired.
Gating strategy	Cells were gatted taking into accout the FMOs and unstained control samples using standart methods for all experiments.

☐ Tick this box to confirm that a figure exemplifying the gating strategy is provided in the Supplementary Information.
