# Supplementary material for: Urothelial organoids originating from Cd49fhigh mouse stem cells display Notch-dependent differentiation capacity
Source: Nat Commun. 2019 Sep 27;10:4407. doi: 10.1038/s41467-019-12307-1 (PMC6764959; doi:10.1038/s41467-019-12307-1)
Supplement: Supplementary file 6 — Description of Additional Supplementary Files [file 41467_2019_12307_MOESM6_ESM.pdf]

Title: Supplementary Movie 1
Description: Example of proliferative and differentiated during FRAP assay (prebleaching, post-bleaching and recovery) over a 14 h period.

Title: Supplementary Data 1.
Description: Differentially expressed genes in P vs D organoids from bulk RNA-Seq analyses.

Title: Supplementary Data 2.
Description: Cluster markers and variable features for individual and integrated scRNA-Seq analyses.
